# Supplementary material for: Relevance of CSF, Serum and Neuroimaging Markers in CNS and PNS Manifestation in COVID-19: A Systematic Review of Case Report and Case Series
Source: Brain Sci. 2021 Oct 14;11(10):1354. doi: 10.3390/brainsci11101354 (PMC8533964; doi:10.3390/brainsci11101354)
Supplement: Supplementary file 1 [file brainsci-11-01354-s001.zip › brainsci-1375303-supplementary.pdf]

Table S1. Studies with data from case reports and case series on COVID-19 associated CNS manifestation.

Author/ country	Age /gender	Onset COVID-19 to Neurological symptom onset	Co-morbidity	Neurological presentation	CSF findings	Oligoclonal bands	Serum/CSF AQP4, and MOG Ab and other ABs	CT findings	MRI findings	Diagnosis	Management	Outcomes	Severity
D.D. Cavalcanti et. al./ USA	41/F	NA	None	Confusion, global aphasia, left gaze preference with NIHSS 16, extensor posturing to noxious stimuli	WBC-41/mm^3 neutrophil 84% Protein: 616 mg/dl Glucose: normal; ***	NA	NA	CT head: venous infarction in the left basal ganglia, thalamus, and mesial temporal lobe with hemorrhagic transformation, intraventricular hemorrhage. CT venogram- occlusion of internal cerebral veins.	NA	Venous sinus thrombosis stroke	EVD, Heparin infusion without bolus treatment	Deceased	Severe
Scullen T. et. al./ USA	63/F	10 days	HTN, Obesity	Altered mental status without focal deficit	WBC<5/mm^3 Protein: normal Glucose: normal, ***	NA	NA	Hypodensities within bilateral globus pallidi as well as a focal parenchymal hemorrhage in the left occipital pole	FLAIR changes at the same locations and diffusion restriction in bilateral globus pallidi and bilateral centrum semiovale	Encephalopathy	NA	NA	Severe
Zoghi A. et. al./ Iran	21/M	14 Days	None	Paresis in lower limbs,	WBC- 150 /mm^3(60%	Neg	Neg	NA	MRI of the cervical and thoracic spine	ADEM and LETM	PLEX x 5 days,	Improved	Non-severe

				paresthesia in lower limbs, urinary retention, drowsy	lymphocytes). Protein 281mg/dl Glucose: 34mg/dl, serum glucose110 mg/dl SARS-CoV-2 PCR: neg				LETM with an > 3 segments in the spinal cord. Brain MRI showed bilateral long corticospinal tract lesions in internal capsules extending to the cerebral peduncles and pons. Hyperintensity in the splenium of the corpus callosum.		Empiric Tx with vancomycin, meropenem and Acyclovir		
Guennec L.L. et. al./ France	69/M	5 Days	DM, HTN, seizure	Status epilepticus	WBC < 5/ mm^3 Protein: 66mg/dl Glucose: 105mg/dl, serum glucose 360 mg/dl SARS-CoV-2 PCR: neg	NA	NA	Unremarkable	Hyperintensity of the right orbital prefrontal cortex adjacent to the olfactory bulb, which seemed to spread toward the right mesial prefrontal cortex and to the right caudate nucleus	Non-convulsive status epilepticus	IVIG	Improved	Severe,
Cani I. et. al./ Italy	77/F	18 Days	None	Impaired consciousness, stimulus induced myoclonus, positive	WBC<5/ mm^3 Protein: 56mg/dl Glucose: **; ***	Negative	NA	NA	Diffuse white-matter lesions consistent with chronic small vessel disease	Encephalopathy	IVMP x 60mg x 10 days	Improved	Non - severe

				primitive reflexes	SARS-CoV-2 PCR: neg				without contrast enhancement				
Benameur K. et. al./ USA	31/F	5 Days	Sickle cell disease	Altered mentation	WBC: 115/ mm^3 51% Neutrophils, Protein: >200mg/dl Glucose: 40 mg/dl; *** SARS-CoV-2 PCR: neg SARS-CoV-2 CSF IgM pos	NA	NA	NA	Occlusive thrombus in the right internal carotid artery with associated restricted diffusion on DWI and edema on T2/FLAIR in the right cerebral hemisphere consistent with infarct. Foci of T2 signal abnormality were also identified within the cervical spinal cord	Acute infarct, Encephalitis, Myelitis	Hydroxychloroquine and Peramivir 100mg Daily	Deceased	Severe
Benameur K. et. al./ USA	34/M	9 Days	HTN	Profound encephalopathy, multifocal myoclonus.	WBC: $1/mm^3$ Protein: 37 mg/dl Glucose: 111mg/dl; *** SARS-CoV-2 PCR: neg SARS-CoV-2 CSF IgM pos	N A	NA	NA	Non-enhancing hyperintense lesion within the splenium of the corpus callosum on FLAI and DWI	Encephalopathy	NA	NA	Severe

Benameur K. et. al./ USA	64/M	NA	HTN	Profound encephalopathy, multifocal myoclonus.	WBC < 5/ mm^3 Protein: 21mg/dl Glucose: 88mg/dl, *** SARS-CoV-2 PCR: neg SARS-CoV-2 CSF IgM pos	NA	NA	NA	Hyperintense area in the right temporal lobe	Encephalopathy	NA	Improved	Severe
Dogan L. et. al./ Italy	49/M	NA	None	NA	WBC: 0 Protein: 37.6mg/dl Glucose: 130mg/dl; *** SARS-CoV-2 PCR: neg	NA	NA	NA	Bilateral frontal cortical hyperintensity together with focal effacement of right frontal sulci. DWI showed frontal cortical hyperintensity and leptomeningeal enhancement.	Meningoencephalitis	Lopinavir/Ritonavir, Azithromycin, Hydroxychloroquine, Favipiravir PLEX x 6 cycles	Improved	Severe
Dogan L. et. al./ Italy	59/M	NA	HTN	NA	WBC: 0, Protein: 73.2mg/dl Glucose: 201mg/dl; *** SARS-CoV-2 PCR: neg	None	NA	NA	MRI bilateral extensive hyperintensity frontal and parietal white matter. DWI images showed matching areas restrictions and leptomeningeal enhancement	Meningoencephalitis	Azithromycin, Hydroxychloroquine, Favipiravir PLEX x 9 cycles	Improved	Severe

Dogan L. et. al./ Italy	59/M	NA	HTN, DM, Obesity	NA	WBC: 0 Protein: 65.7mg/dl Glucose: 121mg/dl; *** SARS-CoV-2 PCR: neg	None	NA	NA	Normal	Meningoencephalitis	Azithromycin, Hydroxychloroquine, Favipiravir PLEX x 1 cycle	Deceased	Severe
Dogan L. et. al./ Italy	51/F	NA	HT, DM	NA	WBC: 0 Protein: 131mg/dl Glucose: 120 mg/dl; *** SARS-CoV-2 PCR: neg	None	NA	NA	Normal	Meningoencephalitis	Azithromycin, Favipiravir PLEX x 5 cycles	Patient regained consciousness after 1st cycle	Severe
Dogan L. et. al./ Italy	55/M	NA	HTN	NA	WBC: 0 Protein: 52mg/dl Glucose: 67mg/dl; *** SARS-CoV-2 PCR: neg	None	NA	NA	Normal	Meningoencephalitis	Azithromycin, Hydroxychloroquine, Favipiravir<br><br>PLEX x 5 cycles	NA	Severe
Dogan L. et. al./ Italy	22/M	NA	Autism	NA	WBC: 0 Protein: 57mg/dl Glucose: 59mg/dl; ***	None	NA	NA	NA	Meningoencephalitis	AZI, HC, FAV PLEX x 3 cycles	NA	Severe
Virhammar J. et. al. / Sweden	55/F	7 Days	NA	Lethargic, stuporous, Multifocal Myoclonus	WBC: <5/mm^3 Protein: Normal Glucose: **; ***	present	NA	Symmetrical hypodensities in the thalami. and midbrain	MRI hyperintensity in subinsular regions, medial temporal lobes, hippocampi, and cerebral	AHNE	IVIG followed by PLEX	Improved	Severe

					SARS-CoV-2 PCR: pos SARS-CoV-2 CSF IgG				peduncle, pons and SWI small foci of petechial hemorrhage in central thalami and subinsular regions				
Farhadian S. et. al./ USA	78/F	NA	Immunocompromised, renal transplanted patient	Altered mental state, bilateral tremor of upper and lower extremity	WBC < 5/ mm^3 Protein: 43mg/dl Glucose: **, *** SARS-CoV-2 PCR: neg	None	None	NA	Atrophy and patchy periventricular and subcortical white matter hyperintensities, which were interpreted as sequelae of small vessel ischemic disease	Encephalopathy	Hydroxychloroquine, tocilizumab	Improved	Non-severe
Pinto A.A. et. al./ USA	44/F	7 Days	NA	Mild expressive and receptive aphasia, visual and sensory inattention, paresis in right upper and lower limbs	WBC - 13/mm^3 Protein: 50.7mg/dl Glucose 29mg/dl, *** SARS-CoV-2 PCR: neg	Absent	Anti-MOG: positive	NA	T2-hyperintensity in periventricular, along the left temporal and occipital horns and into the subcortical deep white matter	Anti-MOG syndrome	IVMP 1g x 5 days followed by oral prednisolone 60mg daily. PLEX x 5 cycles	Improved.	Non-Severe
Espíndola OM et.al. /Brazil	NA	NA	NA	NA	WBC-18/mm3, protein: 60 mg/dl, glucose: 43, ***	NA	NA	NA	NA	Encephalitis	NA	NA	NA

					SARS-CoV-2 PCR: neg								
Espíndola OM et.al. /Brazil	NA	NA	NA	NA	WBC- 2/mm3, Protein: 23 mg/dl, Glucose: 96, *** SARS-CoV-2 PCR: neg	NA	NA	NA	NA	Encephalitis	NA	NA	NA
Espíndola OM et.al. /Brazil	NA	NA	NA	NA	WBC- 3/mm3, Protein: 51 mg/dl, Glucose: 115, *** SARS-CoV-2 PCR: neg	NA	NA	NA	NA	Delirium	NA	NA	NA
Espíndola OM et.al. /Brazil	NA	NA	NA	NA	WBC- 3/mm3, Protein: 51 mg/dl, Glucose: 115, *** SARS-CoV-2 PCR: neg	NA	NA	NA	NA	Delirium	NA	NA	NA
Mingxiang Ye. et. al./ China	NA/M	13 Days	NA	Confusion, nuchal rigidity	WBC <5mm^3 Protein: 27mg/dl Glucose: 314mg/dl; *** SARS-CoV-2 PCR: neg	NA	NA	Normal	NA	Encephalitis	Supportive treatment only including Mannitol infusion.	Improved	Non-severe
Filatov A. et. al./ USA	74/M	1 Day	Atrial fibrillation, CVA,	Headache, altered mental	WBC < 5mm^3	NA	NA	Normal except an area of	NA	Encephalopathy	Vancomycin,	NA	Severe

			Parkinson's disease, COPD, recent cellulitis	status, aphasic, apraxia	Protein: 68mg/dl Glucose: 75mg/dl; ***			encephalomalacia in left temporal region consistent with prior stroke			Meropenem, acyclovir along with hydroxychloroquine and lopinavir/ritonavir		
Guilmot A. et. al. /Belgium	80/NA	NA	NA	Paroxysmal dysarthria, tonic-clonic seizure, visual hallucinations, short term memory disturbance and anxiety	WBC > 5mm^3 Protein: 46mg/dl Glucose: **; *** SARS-CoV-2 PCR: neg	Positive OCB	anti-Caspr2 IgG antibodies in serum and in CSF	NA	Normal	Encephalitis	IVMP followed by PLEX (unspecified duration)	Improved	Non-severe
Guilmot A. /et. al./ Belgium	62/NA	16 Days	NA	Aggressiveness, paranoia, temporal status epilepticus	WBC < 5mm^3 Protein: 51mg/dl Glucose: **; *** SARS-CoV-2 PCR: neg	Mirror	NA	Normal	NA	Encephalopathy	NA	NA	Severe
Guilmot A. /et. al. / Belgium	71/NA	5 Days	NA	Delirium, with akathisia, choreiform involuntary movement of upper limbs and gait ataxia.	WBC < 5mm^3 Protein: 32mg/dl Glucose: **; *** SARS-CoV-2 PCR: neg	Negative	Anti-GD1b IgG-high titre	Normal	NA	Encephalopathy	NA	NA	Non-severe

Guilmot A. /et. al. / Belgium	60/NA	7 Days	NA	Delirium	WBC < 5mm3 Protein: 35mg/dl Glucose: ** ;*** SARS-CoV-2 PCR: neg	Negative	Neg	Normal	NA	Encephalopathy	NA	NA	Severe
Guilmot A. /et. al. / Belgium	66/NA	5 Days	NA	Delirium	WBC < 5mm3 Protein: 18mg/dl Glucose: **; *** SARS-CoV-2 PCR: neg	Mirror	NA	Normal	NA	Encephalopathy	NA	NA	Severe
Guilmot A. /et. al. / Belgium	58/NA	21 Days	Intracardiac thrombus	Stroke like symptoms	WBC < 5mm3 Protein: 52mg/dl Glucose: **; *** SARS-CoV-2 PCR: neg	NA	NA	NA	NA	Acute cerebrovascular disease	NA	NA	Severe
Guilmot A. /et. al. / Belgium	74/NA	NA	Patent foramen ovale, DVT	Stroke like symptoms	WBC < 5mm3 Protein: 51mg/dl Glucose: **; *** SARS-CoV-2 PCR: neg	Mirror	NA	NA	NA	Acute cerebrovascular disease	NA	NA	Non-severe

Guilmot A. /et. al. / Belgium	54/NA	NA	Pulmonary embolism	Stroke like symptoms	WBC < 5mm^3 Protein: 11mg/dl Glucose: **; *** SARS-CoV-2 PCR: neg	Negative	NA	NA	NA	Acute cerebrovascular disease	NA	NA	Severe
Krett, J.D. et. al./ Canada	69/M	4 Days	CAD, HTN, DM type 2	Unresponsive and diffusely paretic.	WBC<5mm^3 Protein: >45 mg/dl, Glucose: **, *** SARS-CoV-2 PCR: neg	NA	NA	Unremarkable	MRI: showed multicompartmental hemorrhages with mild surrounding vasogenic edema and no abnormal enhancement. MRI spine: unremarkable	ANE	Hydroxychloroquine x 7 days	Partially improved	Severe
Moriguchi T. et. al./ Japan	24/M	9 Days	None	Altered consciousness, generalized seizure, neck stiffness.	CSF: WBC 12/ mm^3 Protein: NA Glucose: **; *** SARS-CoV-2 PCR: pos	NA	NA	CT head- normal with no evidence of edema	DWI changes in inferior horn of right lateral ventricle. FLAIR hyperintensity in the right mesial temporal lobe and hippocampus with slight hippocampal atrophy. Contrast-enhanced imaging showed no enhancement	Right lateral ventriculitis and Encephalitis	Ceftriaxone, vancomycin, acyclovir, Favipiravir and steroids. Keppra for seizure.	NA	Severe

Franke C. et. al./ Germany	76/M	NA	NA	Downbeat nystagmus, generalized stimulus-sensitive myoclonus	WBC: 1/ mm^3 Protein: 31.4mg/dl Glucose:76mg/dl; ***	Positive	Neg	NA	Normal	Autoimmune encephalitis	NA	NA	NA
Franke C. et. al./ Germany	58/F	NA	NA	Delirium	WBC: 5mm^3 Protein: 36.8mg/dl Glucose:93mg/dl; ***	Positive	Neg	NA	Normal	Autoimmune encephalitis	NA	NA	NA
Franke C. et. al./ Germany	76/M	NA	NA	Right-sided stimulus-sensitive myoclonus	WBC: 177/mm^3 Protein: 93.7mg/dl Glucose:83mg/dl; ***	Positive	Neg	NA	Normal	Autoimmune encephalitis	NA	NA	NA
Franke C. et. al./ Germany	58/F	NA	NA	Right-sided orofacial myoclonus	WBC: 17/mm^3 Protein: 28.1 mg/dl Glucose: 52mg/dl; ***	Negative	Anti-Yo positive	PET-CT showed evidence of florid encephalitis with tracer increase in the basal ganglia and limbic system as well as in the cerebellar region of the inferior cerebellar artery	Normal	Autoimmune encephalitis	NA	NA	NA
Franke C. et. al./ Germany	54/M	NA	NA	Delirium, myoclonus, epileptic seizures	WBC<5mm^3 Protein: 25.6mg/dl	NA	Neg	NA	Marked edema of the fornix.	Autoimmune encephalitis	NA	NA	NA

					Glucose: 75mg/dl; ***								
Franke C. et. al./ Germany	77/M	NA	NA	Right-sided faciobrachial myoclonus,	WBC<5mm^3 Protein:68.2mg/dl Glucose: 145mg/dl;***	Positive	Negative	NA	Ischemic lesion (DWI) changes of the right middle cerebral artery (MCA) region.	Autoimmune encephalitis,infarct	NA	NA	NA
Franke C. et. al./ Germany	48/M	NA	NA	Oculomotor paresis, transient generalized myoclonus, prolonged awakening	WBC<5/mm^3 Protein: 74.1mg/dl Glucose:80mg/dl; ***	Negative	Negative	NA	Normal	Autoimmune encephalitis	NA	NA	NA
Franke C. et. al./ Germany	78/M	NA	NA	Dystonia right > left upper limb, delirium	WBC<5/mm^3 Protein:437m/dl Glucose: 94mg/dl; ***	NA	Negative	NA	Normal	Autoimmune encephalitis	NA	NA	NA
Franke C. et. al./ Germany	75/F	NA	NA	Aphasia, neglect, encephalopathy , stupor, impaired consciousness	WBC<5mm^3 Protein:16.8mg/dl Glucose: **; ***	Negative	Negative	NA	Normal	Autoimmune encephalitis	NA	NA	NA
Franke C. et. al./ Germany	69/M	NA	NA	Downbeat nystagmus, orofacial myoclonus, delirium	WBC: 8/ mm^3 Protein: 59.4mg/dl Glucose: 123mg/dl	Positive	Negative	NA	Normal	NA	NA	NA	NA

Fadakar N. et. al./ Iran	47/M	10 Days	Unremarkable	Vertigo, headache, mild dysarthria, wide based ataxic gait, head titubation, mild truncal swaying, nystagmus	WBC: 10mm³, 60% lymphocytes Protein:58mg/dl Glucose:60mg/dl; *** SARS-CoV-2 PCR: pos	Neg	Neg	NA	Bilateral cerebellar hemispheres as well as vermis hyperintensities and edema with cortical-meningeal enhancement of cerebellum on post contrast images	Acute Cerebellitis	Lopinavir/ritonavir administered 400/100 mg twice daily for 14 days	Improved	Non-severe
Guilmot A. /et. al. / Belgium	62/NA	21 Days	NA	Ophthalmoplegia, palatal myoclonus, neck stiffness and areflexic flaccid tetraplegia, coma	WBC<5mm³ Protein:32mg/dl Glucose: **; *** SARS-CoV-2 PCR: neg	Mirror	Anti-GD1b IgG high titer	NA	Unremarkable	Encephalopathy	IVIG	Partial improvement	Severe
GuilmotA. /et. al./ Belgium	66/NA	21 Days	NA	Delirium, agitation, hallucination, neck stiffness, diffuse myoclonus, bilateral ophthalmoplegia, palatal tremor, apnea and coma	WBC<5mm³ Protein: 45mg/dl Glucose: **; *** SARS-CoV-2 PCR: neg	Neg	Neg	NA	Unremarkable	Encephalopathy	IVIG	Improving	Severe

Guilmot A. /et. al./ Belgium	54/NA	5 Days	NA	Behavioral changes, irritability, paranoia.	WBC<5/mm^3 Protein: 18mg/dl Glucose: **; ***	Mirror	Neg	NA	NA	Encephalopathy	NA	NA	Non-severe
Domingues R.B. et. al./ Brazil	42/F	21 Days	Paresthesia of left upper limb, later progression to left hemithorax and hemiface	None	WBC<5mm^3 Protein: 32mg/dl Glucose: 62mg/dl; *** SARS-CoV-2 PCR: pos	Neg	Neg	NA	Brain MRI normal<br><br>MRI Cervical cord hyperintense lesion spanning multiple levels without enhancement	Transverse Myelitis	NA	NA	Non-severe
Mawhinney J.A. et. al./ UK	41/M	10 Days	Congenital nystagmus	Agitated, severe headache.	WBC<5mm^3 Protein: 19mg/dl Glucose: 48mg/dl; *** SARS-CoV-2 PCR: neg	Neg	Neg	Normal	Normal	NA	Antibiotics and acyclovir and olanzapine	Improved	Severe
Delamarre L. et. al./ France	51/M	21 Days	None	Coma, right sided 6th cranial nerve palsy, rhythmic movement of right upper limb	WBC<5mm^3 Protein: NA Glucose: **; *** SARS-CoV-2 PCR: neg	NA	Negative	Normal	Progressing lesions with diffuse hyperintense lesions in the thalami, cerebellum, brainstem, supratentorial grey and white matters	AHNE	IVMP x 3 days followed by IVIG x 5 days	Improved.	Severe
Grimaldi S. et. al./ France	72/M	17 Days	None	Cerebellar syndrome (tremor,	WBC<5mm^3 Protein: 49mg/dl	Negative	Negative	NA	Normal	Autoimmune Encephalitis	IVIG x 5 Days followed by	Improved	Non-severe

				ataxia, dysarthria) with stimulus-sensitive diffuse myoclonus	Glucose: **; *** SARS-CoV-2 PCR: neg						IVMP x 5 days.		
Zambreanu L. et. al. / UK	66/F	18 Days	None	Confusion, generalized tonic-clonic seizure	WBC<5mm³ Protein:100mg/dl Glucose: 35mg/dl; *** SARS-CoV-2 PCR: neg	Negative	Neg	Normal	MRI showed symmetrical hyperintensities in mesial temporal lobes and medial thalami and to a lesser extent upper pons, as well as scattered subcortical white matter hyperintensities.	Limbic encephalitis	IVMP x 3 days followed by IVIG (unspecified time)	Improved	Non-severe
Matos A.R. et. al./ Portugal	42/M	7 Days	None	Confusion, hypokinesia, apathy, hyposmia, dysexecutive syndrome, perseveration, dysphonia and dysphagia	WBC<5mm³ Protein: 78mg/dl Glucose: **; *** SARS-CoV-2 PCR: neg	NA	NA	Multiple hypodense lesions involving the white matter, basal ganglia, and thalami.	Multiple hyperintense lesions involving the deep and subcortical white matter on both hemispheres, as well as the thalami, basal ganglia, and basal pons; some showed restricted diffusion on DWI	CNS vasculopathy	IVIG followed by IVMP x 5 days	Improved	Non-severe
Byrnes S. et. al./ USA	36/M	8 Days	Polysubstance abuse	Slurred speech,	WBC: >5/mm3	NA	N A	Normal	Multiple focal enhancing lesions the	Encephalopathy	IVMP followed by	Partially Improved	Non-severe

				pinpoint pupils, agitation, choreiform movement	lymphocyte predominance Protein: increased Glucose: **; ***				bilateral medial putamen and left cerebellum. There were also several cortical and subcortical lesions including the hippocampus, primarily on the left side, along with punctate restricted diffusion in the right basal ganglia.		IVIG x 5 days		
Goodloe TB 3rd et. al./ USA	52/M	0 Days	DM, HTN, CAD	Altered mental status	WBC 0/m^3 Protein:46mg/dl Glucose: 121mg/dl; ***	NA	NA	CT head no acute changes	No acute changes	Encephalopathy	NA	Improved	Non-severe
Elkady A. et. al./ EGYPT, USA	33/F	4 Days	None	Coma and status epilepticus	WBC- 26/ mm^3 90 % lymphocytes Protein: 541mg/dl Glucose: normal; ***	Neg	NA	Diffuse brain edema with right thalamic and right cerebellar hemorrhages.	Bilateral hyperintensity in thalami and cerebellum with hemorrhagic components and rim contrast enhancement within brain; left occipital minimal leptomeningeal enhancement	AHNE	High dose IVMP	Deceased	Severe
Chalil A. et. al./ Canada	48/F	15 Days	None	Coma, with absent pupil and corneal reflex	WBC: 76/mm^3, 65% Neutrophils Protein: NA	NA	N A	Head CT with CT angiography demonstrated extensive bilateral parietal and	Gadolinium enhancement with hyper-intense signal surrounding the hemorrhages, in	AHNE	Hydroxychloroquine and Tocilizumab	The patient was extubated with severe	Severe

					Glucose: **; *** SARS-CoV-2 PCR: neg			occipital intraparenchymal hemorrhage with intraventricular extension and acute hydrocephalus	keeping with vasogenic edema previously seen on CT			neurological deficits	
Guillan M. et. al./ Spain	67/ M	15 Days	HTN, smoking and Alcohol consumption	Confused, gait ataxia, temporo-spatial disorientation, dysarthria, partial cortical blindness and anosognosia with visual confabulation, optic ataxia, difficulty in visual scanning, simultagnosia	WBC: 30/ mm^3, lymphocytes 90%, Protein: 314.1mg/dl Glucose: normal; ***	Mirror	NA	Bilateral parietooccipital and right cerebellar hypoattenuating lesions with areas of cortical hyperattenuating involvement.	Arterial ischemic lesions in the right MCA, the left PCA, and a segment of the right superior cerebellar artery (SCA) with cortical laminar necrosis. The MR angiography normal	CVA	Hydroxychloroquine, ceftriaxone and azithromycin	Improved	Non-severe
Espinosa P.S. et. al./ USA	72/M	6 days	HTN, hyperlipidemia, Type 2 DM	Coma	WBC: <5/mm3 Protein: 27 mg/dl Glucose: 87; ***	NA	NA	NA	Left parietal infarct DWI and FLAIR hyperintense changes in left parietooccipital region	Encephalopathy/infarct	Hydroxychloroquine, ceftriaxone, azithromycin	Not improved	Severe
Giorgianni A. et. al./ Italy	22/F	15 Days	Type 1 DM	Acute flaccid tetraparesis,	WBC: NA Protein: 53mg/dl	NA	NA	Tiny right frontal parenchymal hemorrhage	Late subacute tiny frontal hemorrhage MRI spine NA	Transverse Myelitis	NA	Improved, discharged	Severe

				hyperreflexia, migrant hypoesthetic and dysesthetic manifestations in lower limbs, fecal and urinary incontinence.	Glucose: 139mg/dl; serum 396mg/dl							to rehab after 30 days	
Abdi S. et. al./ Iran	58/M	30 Days	None	Decreased consciousness, gait ataxia	WBC: 0/mm^3 Protein: 15mg/dl Glucose: 105mg/dl; *** SARS-CoV-2 PCR: neg	Negative	NA	NA	MRI diffuse hyperintensity, particularly at the left-side without enhancement, involvement of cortical as well as deep gray matter, and dorsal midbrain	ADEM	IV dexamethasone 8mg TDS	Deceased	Severe
Kakadia B. et. al./ USA	69/M	NA	HTN	Disorientation, inattention, bradyphrenia	WBC: <5mm^3 Protein Normal Glucose: normal; *** SARS-CoV-2 PCR: neg SARS-CoV-2 CSF IgG	NA	NA	NA	MRI restricted diffusion and hyperintensity in the splenium of the corpus callosum	MERS	NA	Improved	Non-severe
Djellaoui A. et. al./ France	69/F	NA	CAD, endometrial cancer, right breast cancer.	Generalized seizures, mutism delirium, asthenia	WBC: <5mm^3 Protein: 25mg/dl Glucose: **; ***	Neg	Neg	NA	Symmetric hyperintensities located mainly in the temporal and occipital lobes with	PRES	Antiepileptic drugs	Improved	Non-severe

					SARS-CoV-2 PCR: neg				increased ADC values and leptomeningeal enhancement in the same areas in gadolinium sequence				
Morvan A. et. al./ France	56/M	NA	Malnutrition, nephrolithiasis with left renal abscess	Coma	WBC: < $5mm^3$ Protein: 79mg/dl Glucose: 45; *** SARS-CoV-2 PCR: neg	NA	NA	Acute hydrocephalus with diffuse cerebral edema, bilateral thalamic hyperdensities concerning for hemorrhage with discrete contrast enhancement	Compression of the 3rd ventricle by both thalami causing hydrocephalus, compression of the 4th ventricle by the cerebellum, diffuse signs of intracranial hypertension and a starting tonsillar engagement	AHNE	NA	Deceased	Severe
Forestier G. et. al./ France	55/M	NA	None	Headache, dizziness, impaired consciousness	WBC< $5mm^3$ Protein: 46mg.dl protein Glucose: normal; ***	NA	NA	NA	Increased diffusion weighted signal in the splenium of the corpus callosum	Cytotoxic lesion of corpus callosum	NA	Improved.	Severe
Munz M. et. al./ Germany	60/M	8 Days	HTN	Bladder dysfunction, weakness of lower limbs. Hypesthesia below Th9 level and spastic paresis	WBC-27/ mm^3 Lymphocytes Protein: 117.7mg/dl Glucose: **; *** SARS-CoV-2 PCR: neg	Negative	NA	NA	MRI spine- Patchy hyperintensity at Th9-10 and at Th3-5 level. MRI brain- normal	Transverse Myelitis	Methylprednisolone 100mg/d	Improved	Non-severe

Vaschetto R. et. al./ Italy	64/M	16 Days	HTN	Coma, tetraplegia	WBC < 5mm^3 Protein: 91.5mg/dl Glucose: 117mg/dl; *** SARS-CoV-2 PCR: neg	NA	Neg	Cortical-subcortical blood-related hyperdensities in the bilateral fronto-parietal and right occipital lobes.	Signal restriction in parietal and parieto-occipital region and at the pons as well as scattered regions of hemorrhage	CNS vasculitis	IVIG x 5 days then IVMP x 5days	Mildly improved	Severe
Zang T. et. al./ USA	40/F	9 Days	HTN, dyslipidemia	Dysphagia, dysarthria, expressive aphasia and mild facial droop	WBC: < 5/ mm^3 Protein: normal Glucose: normal; *** SARS-CoV-2 PCR: neg	NA	NA	Multifocal patchy areas of white matter hypoattenuation	Extensive patchy hyperintensity bilateral frontoparietal white matter, anterior temporal lobes, basal ganglia, external capsules, and thalami. some of these foci demonstrated DWI changes with questionable minimal enhancement	ADEM	Hydroxychloroquine and IVIG x 5 days	Improved	Severe
Cariddi L.P. et. al./ Italy	64/F	25 days	HTN, GERD, hyperuricemia, dyslipidemia, OSA and paroxysmal AF	Altered mental status, decreased left nasolabial fold, paresis in lower limbs, global hyporeflexia.	WBC: <5mm3 Protein: 53 mg/dl Glucose: 139; *** SARS-CoV-2 PCR: neg	NA	Neg	Posterior frontal and temporo-parieto-occipital symmetric bilateral hypodensity of the subcortical white matter, and a tiny left occipital parenchymal hemorrhage	FLAIR image shows that vasogenic edema is reduced but still detectable and T2 Gradient-Echo reveals right temporal hypodensity, correlated to hemorrhagic process	PRES	Hydroxychloroquine	Partially improvement	Severe

Delorme C. et. al./ France	72/ M	15 Days	None	Psychomotor agitation, cognitive and behavioral frontal lobe syndrome, upper limbs myoclonus, cerebellar ataxia	WBC: 6mm^3 Protein:23 mg/dl Glucose: **; *** SARS-CoV-2 PCR: neg	Negative	NA	Brain FDG-PET/CT imaging showed bilateral prefrontal and left-sided parieto-temporal hypometabolism and a slight hypermetabolism within the cerebellar vermis.	Unremarkable	Encephalitis	IVIG (duration not specified)	Improved	Non-severe
Delorme C. et. al./ France	66/F	7 Days	None	Psychomotor slowing, Cognitive and behavioral frontal lobe syndrome	WBC: 1mm^3 Protein:30 mg/dl Glucose: **; *** SARS-CoV-2 PCR: neg	Negative	NA	Brain FDG-PET/CT marked hypometabolism within the bilateral prefrontal and associative posterior cortices and hypermetabolism within the bilateral striatum and the cerebellar vermis	Non-specific white matter hyperintensities.	Encephalitis	IVIG x 5 days followed by IV pulse corticosteroids	Improved	Non-severe
Delorme C. et. al./ France	60/F	Same day	Temporal lobe epilepsy (hippocampal sclerosis)	Acute anxiety, depressed mood, akathisia, gait imbalance, psychomotor agitation, dysexecutive syndrome,	WBC: <5mm^3 Protein:25 mg/dl Glucose: **; *** SARS-CoV-2 PCR: neg	Negative	NA	Brain FDG-PET/CT showed hypometabolism within the bilateral orbitofrontal cortices, and a slight hypermetabolism in the bilateral striatum and cerebellar vermis.	Right mesial sclerosis	Encephalitis	IV pulse corticosteroids x 3 days Antidepressants	Improved	Non-severe

				cerebellar ataxia								
Delorme C. et. al./ France	69/M	7 Days	HTN, Type II DM	Generalized convulsive Status epilepticus	WBC: <5mm^3 Protein:66 mg/dl Glucose: **; *** SARS-CoV-2 PCR: neg	Negative	NA	Brain FDG-PET/CT showed hypometabolism within the bilateral prefrontal and associative posterior cortices, and hypermetabolism within the cerebellar vermis.	Right orbitofrontal hyperintensities on T2-weighted image	Encephalitis	Antileptics. IVIG x 5 days. Pulse corticosteroids x 5 days	Improved	Severe
Afshar H. et. al./ Iran	39/F	10 Days	NA	Drowsiness, generalized tonic-clonic seizure	WBC: <5/ mm^3 Protein: 19mg/dl Glucose: 61mg/dl SARS-CoV-2 PCR: neg	Neg	Neg	NA	FLAIR high signal intensities in bilateral thalami, medial temporal and pons without gadolinium enhancement	Encephalitis	Levetiracetam 500mg IVIG followed by IVMP x 6 days	Improved	Severe
Novi G. et. al./ Italy	64/F	15 Days	Vitiligo, HTN, MGUS	Irritability, severe vision loss, sensory deficit in right leg, anosmia, ageusia	WBC: 22/mm^3 (mainly lymphocytes) Protein: 45.2mg/dl Glucose: **; *** SARS-CoV-2 PCR: pos	Mirror	Neg	NA	Multiple T1 post-Gd enhancing lesions of the brain, associated with a single spinal cord lesion at the T8 level and with bilateral optic nerve enhancement	ADEM	IVMP x 5 days IVIG x 5 days	Partial Improvement	Non-severe
Hayashi M. et. al./ Japan	75/ M	NA	Alzheimer's disease	Altered mental status,	NA	NA	NA	NA	Abnormal hyperintensity in the	MERS	Favipiravir, pulse	Deceased	Severe

				hand tremors, urinary incontinence.					splenium of corpus callosum (SCC) on diffusion-weighted image.		corticosteroid, ciclesonide and meropenem		
Yong M.H. et. al./ Singapore	61/M	20	HTN, hyperlipidemia, DM	Flaccid tetraplegia	NA	NA	NA	NA	Increased symmetrical FLAIR signal throughout the white matter and thalamus.<br>SWI evidence of microhemorrhages	AHLE	IVIG then IVMP x 5days	Partially improved	Severe
Haqiqi A. et. al./ UK	56/M	7 Days	HTN, CKD, hypercholesterolemia, asthma, pre-obesity	Coma	WBC <5/mm^3 Protein: 71mg/dl Glucose: 43mg/dl, serum 86mg.dl SARS-CoV-2 PCR: neg	Positive	NA	Diffuse white matter hypodensity and multiple bilateral white matter hemorrhagic foci involving the corpus callosum	Increased symmetrical FLAIR signal throughout the white matter. There are also some cystic hemorrhagic areas within both cerebral hemispheres. There are some areas of restricted diffusion within the white matter	AHLE	Conservative management	Improved	Severe
Varadan B. et. al./ India	46/M	35 Days	Alcoholic CLD	Headache, altered mental status, left facial nerve palsy, paresis in left upper and lower limbs	WBC: >5/mm^3 Protein: Increased Glucose: **; ***	NA	NA	Multifocal non-hemorrhagic lesions in both cerebral hemispheres and the brainstem	Hyperintensity bilateral frontal, parietal<br>lobes, left thalamus, left cerebral peduncle, and medulla. patchy areas of rim enhancement	AHLE	5 days of IVMP and antiedema management	Deceased	Severe

									within most of the lesions and DWI changes. Few microbleeds were seen within this lesion.				
Gosh R. et. al./ India	44/F	5 Days	None	Confused, disoriented, memory and thought disorder, generalized tonic-clonic seizure	WBC: 20/mm^3, 90% lymphocytes Protein: 60g/dl Glucose: 70mg/dl, ***	Neg	Neg	NA	T2- hyperintensity left fronto-parietal and right posterior parietal areas with and signal blooming in gradient recalled echo (GRE)	AHNE	IVMP x 5 days	Deceased	Severe
Memon A.C. et. al./ USA	65/ F	56 Days	Borderline diabetes and obesity	Lhermitte's phenomenon, urinary incontinence, sensory level at T10	WBC: 20/mm^3, 91% lymphocytes Protein: 81.6g/dl Glucose: 58mg/dl, ***	Neg	Neg	NA	MRI small lacunar infarct at right lateral pontine region Repeat brain MRI T2-signal changes bilateral cortical spinal tracts affecting the posterior limbs of internal capsules to the cerebral peduncles and pons. Repeat MRI cervical spine show multifocal T2-signal C2-C6 without associated enhancement	Late onset Rapidly Progressive Myelitis	Pulse IVMP x 5 days, PLEX x 5 days	Improved	Non-severe
Baghbanian S.M. et. al./ Iran	53/F	15 Days	HTN, DM, Ischemic heart disease	Asymmetric hypotonic paraparesis, areflexia, sensory level	WBC :13/mm^3 cells, predominantly lymphocytes,	Neg	Negative	NA	Brain MRI normal. MRI spinal cord showed longitudinally extensive transverse	Acute transverse myelitis	Corticosteroid and PLEX (unspecified duration)	Improved	Non-severe

				at T11-T12 to pinprick testing, impaired proprioception	Protein: Normal Glucose: normal; *** SARS-CoV-2 PCR: neg				myelitis in the T8-T10 cord segment				
Fumery T. et. al./ Belgium	38/F	15 Days	None	Paresis of lower limbs, hypoesthesia, bladder dysfunction, decreased sensation below T4 level, urinary retention	WBC: 337/ mm3 lymphocytic, Protein: 78mg/dl Glucose: **; ***	Neg	Neg	NA	Hyperintense longitudinal signal involving C3-C4, no gadolinium enhancement	Acute transverse myelitis	IVMP x 8 days	Improved	Non- severe
Franceschi A.M. et. al./ USA	48/M	21 Days	None	Altered mental status	NA	NA	NA	Focal vasogenic/cytotoxic edema in the posterior parietooccipital regions bilaterally, which was subcortical in distribution, with a small right-sided hemorrhage	Vasogenic edema in the posterior parieto-occipital regions with subacute blood products. SWI petechial hemorrhages diffusely distributed throughout the corpus callosum	PRES	None	Improved	Severe
Franceschi A.M. et. al./ USA	67/F	NA	HTN, DM, CAD, gout, asthma	Altered mental status, lethargy, confusion	NA	NA	NA	Edema in the bilateral parietooccipital regions with associated mass effect and	Multiple areas of restricted diffusion with associated edema, in the posterior parieto-	PRES	None	Improved	Non-severe

								cortical sulcal effacement.	occipital. SWI extensive superimposed hemorrhages in the parieto-occipital region along with abnormal enhancement.				
Abdelhady M. et. al./ Qatar	52/M	3 Days	DM type II, G6PD deficiency	Flaccid paralysis and urinary retention	WBC:>5/ mm3 Lymphocytes Protein: increased Glucose: **; *** SARS-CoV-2 PCR: neg	NA	NA	NA	Brain MRI was normal, but the spinal cord MRI displayed a continuous long segment of T2WI hyperintensity in the ventral horns of grey matter in the upper and mid-thoracic cord with no intervening normal cord	Acute Myelitis	Steroids and acyclovir	Deceased	Severe
AlKetbi V. et. al./ UAE	32/M	2 Days	None	Bilateral lower limb paresis and hypotonia, urinary retention	NA	NA	Neg	NA	Extensive diffuse hyperintense signal involving predominantly the grey matter of the cervical, thoracic spinal cord.	Acute Transverse Myelitis	Pulse IVMP x 5 days	Improved	Severe
Chakraborty U. et. al./ India	59/M	NA	None	Ascending flaccid paraplegia, hypotonia of both	WBC< 5/ mm^3 Protein: 71.4mg/dl	NA	NA	NA	MRI spine revealed hyperintensity in the spinal cord at	Acute transverse myelitis	IVMP x 1 day	Deceased	Severe

				lower limbs, areflexia in lower limbs, diminished sensation below T10 level.	Glucose: 75mg/dl; *** SARS-CoV-2 PCR: neg				T6–T7 vertebral level				
Chow C.C.N. et. al./ Australia	60/M	18 Days	HTN, hyperlipidemia, Ex-smoker	Bilateral lower limb paresis, urinary retention, constipation, hypertonia, hyperreflexia, reduced proprioception of lower limb	WBC<5/ mm^3 Protein: 79mg/dl Glucose: 32mg/dl; *** SARS-CoV-2 PCR: neg	NA	Neg	NA	MRI spine demonstrated a long segment of T2 signal from T7 to T10, without significant enhancement	Acute transverse myelitis	IVMP x 3 days	Improved	Non-severe
McCuddy M. et. al./ USA	37/F	22	DM II, HTN, obesity, Pregnancy at 30 weeks	Paresis in upper and lower limbs, hyperreflexia	WBC: <5/ mm^3 Protein: 95mg/dl Glucose: 85mg/dl; *** SARS-CoV-2 PCR: neg	Neg	NA	NA	Multiple T2 hyperintense lesions with restricted diffusion involving the corpus callosum, bilateral cerebral white matter, right pons and in the bilateral medulla and some enhancement	ADEM	Decadron 20mg IV x 5 days and 10 mg x 5 days, PLEX	Improved	Severe
McCuddy M. et. al./ USA	56/M	20	DM II, CKD stage 3, asthma	Coma, leftward eye deviation, hyporeflexia	WBC: <5/ mm^3 Protein: 55mg/dl Glucose: 112mg/dl; ***	Neg	NA	NA	Several T2 hyperintense lesions, many restricted diffusion in cerebral white matter as well as deep cerebellum.	ADEM	IVMP x 5 days then IVIG x 3 days, PLEX	Partial improvement	Severe

					SARS-CoV-2 PCR: neg								
McCuddy M. et. al./ USA	70/F	16	DM II, HTN, HLD, CKD stage 2, obesity, peripheral neuropathy, glaucoma	Coma	WBC: <5/ mm^3 Protein: 63mg/dl Glucose: 87mg/dl; *** SARS-CoV-2 PCR: neg	Present	NA	NA	Several T2 hyperintense lesions, most restricted diffusion, in deep white matter and corpus callosum as well as left brachium spared. Minimum enhancement and no hemorrhage	ADEM	IVMP x 5 days, IVIG x 3 days and PLEX	Partial improvement	Severe
Sarma D. et. al./ USA	28/F	7 Days	Hypothyroidism	Back pain, paresthesia in lower extremities, numbness on tip of tongue, urinary retention, T5 sensory level	WBC: 125/ mm^3 lymphocytes, Protein: 60mg/dl Glucose: normal; *** SARS-CoV-2 PCR: neg	NA	NA	NA	MRI cervical, thoracic spine w/wo contrast revealed elongated signal changes throughout the spinal cord to the conus medullaris and involving the medulla	Acute transverse myelitis	Prednisolone and PLEX x 2 cycles	Improved	Non-severe
Sotoca J. et. al./ Spain	69/W	8 Days	None	Right facial, left hand hypoesthesia, subtle hand weakness	WBC 75/mm^3 (98% lymphocytes) Protein: 283mg/d Glucose: normal; *** SARS-CoV-2 PCR: neg	Neg	Neg	NA	MRI Spinal cord: T2-hyperintensity extending from the medulla oblongata to C7, diffuse patchy enhancing lesions Repeat MRI: transversally and caudally progression until T6	Acute necrotizing Myelitis	IVMP x 5 days followed by PLEX x 5 days	Improved	Non-severe

									level with similar enhancement				
Utukuri P.S. et. al./ USA	44/M	NA	NA	Lethargy, urinary retention, lower limb paresis and numbness, dysarthria, bilateral arm weakness	WBC: 6/ mm^3 ,92% lymphocytes Protein: 36mg/dl Glucose: ** ; *** SARS-CoV-2 PCR: neg	Neg	NA	Normal	MRI Brain several periventricular and juxtacortical lesions Homogeneous brisk enhancement associated with the dominant left parietal lobe juxtacortical/cortical lesion.<br><br>Non-enhancing T2 hyperintense lesions throughout the cervical and thoracic spine	ADEM	IVMP followed by IVIG (duration not reported)	Improved	Non-severe
Valiuddin H. et. al./	61/F	7 days	None	Bilateral upper and lower limb paresis, ankle numbness and tingling in hands and feet up to level of abdomen, urinary retention and constipation	WBC: 3/mm^3 lymphocytes Protein: 87mg/dl Glucose: **; *** SARS-CoV-2 PCR: neg	Negative	Neg	NA	Extensive intramedullary disease throughout the entire length of the cervical spinal cord without pathological contrast enhancement.	Acute transverse myelitis	IVMP x 5 days then PLEX x 5 cycles	Partial improvemen	Non-severe
Durrani M. et. al./ USA	24/M	9 Days	None	Bilateral lower limb paresis,	WBC:>5/mm3 lymphocytic Protein: Normal	Neg	Neg	NA	The MRI showed a non-enhancing T2-weighted hyperintense spanning T7-	Acute Transverse Myelitis	IVMP (duration not reported)	Improved	Non- severe

				overflow urinary incontinence,	Glucose: normal; *** (Values not reported) SARS-CoV-2 PCR: neg				T12 level				
Parsons T. et. al./ USA	51/F	18 Days	NA	Coma, left oculocephalic response impaired	WBC < /5mm^3 Protein: 62mg/dl Glucose: 56mg/dl; *** SARS-CoV-2 PCR: neg	Neg	Neg	CT angiogram normal	FLAIR hyperintensity in deep hemispheric and juxtacortical white matter with hyperintense on DWI and small amount of intraventricular hemorrhage (IVH)	ADEM	IVM x 5 Days followed by IVIG x 5 days	Improved	Severe
Assunção F.B. et. al./ Brazil	49/M	NA	NA	Delayed recovery from sedation, 5 days after extubation	NA	NA	NA	NA	FLAIR multiple hyperintensities deep and periventricular cerebral white matter, splenium of the corpus callosum, and pons. All lesions show restricted diffusion on DWI sequences.	ADEM	NA	NA	Severe
Poyiadji N. et. al./ USA	58/F	3 Day	NA	Altered Mental Status	Limited due to traumatic lumbar puncture	NA	NA	Symmetric low attenuation within the bilateral medial thalami with normal CT angiogram and CT venogram	Hemorrhagic rim-enhancing lesions within the bilateral thalami, medial temporal lobes, and sub-insular regions	AHNE	IVIG	NA	Non- severe

Langley L. et. al./ UK	53/M	NA	NA	Coma, globally hypotonic and areflexia	WBC < /5mm^3 Protein: reported as normal Glucose: **; *** SARS-CoV-2 PCR: neg	Positive	Neg	NA	MRI of the brain and orbits showed multiple hyperintense lesions within the subcortical and deep white matter of the frontoparietal lobes bilaterally. SWI evidence of parenchymal microhemorrhages	ADEM	IVMP x 3 days and tapering prednisone	Partial improvedment	Severe
Dixon, L. et. al./ UK	59/F	10 Days	Aplastic anemia	GTCS, reduced consciousness	WBC: <5/mm^3 Protein: 230mg/dl Glucose: **; *** SARS-CoV-2 PCR: neg	NA	NA	CT showed increased hypodensity and swelling of the brain stem, and a new area of cortical and subcortical hypodensity in the left occipital lobe	Extensive, symmetrical changes throughout the supratentorial and infratentorial compartments. There was diffuse swelling and hemorrhage in the brain stem and both amygdalae	AHNE	High dose dexamethasone	Deceased	Severe
Rasmussen C. et. al./ USA	66/F	19 Days	Pulmonary sarcoidosis, CAD, DM II, HTN, HLD	Right sided weakness, aphasic	NA	NA	NA	Hypodensities within the corpus callosum.	Multiple areas of diffusion restriction within the corpus callosum, corona radiata, and centrum semiovale, with associated T2-FLAIR hyperintensities	CLOCC	NA	Partially improved	Severe

Elkhaled W. et. al./ Qatar	23/M	2 Days	None	Auditory hallucination, restlessness, suicidal ideation, altered sensorium, disorientation and delayed verbal response	WBC <5 /mm^3 Protein: normal Glucose: normal; ***	NA	NA	CT head massive intracranial hemorrhage with diffuse brain edema along with subfalcine and transtentorial herniation.	Brain MRI revealed an isolated oval-shaped lesion in the splenium of corpus callosum	CLOCC	Dexamethasone, Favipiravir	Deceased	Severe
Agarwal N. et. al. /Italy	73/M	21 Days	Altered mental status	None	WBC < 5 /mm^3 Protein 38mg/dl Glucose: 36mg/dl SARS-CoV-2 PCR: neg	Neg	NA	Hypodense lesion within the splenium with mild mass effect on the medial wall of the lateral ventricle	Isolated lesion in the splenium, with a longitudinal	CLOCC	NO IVIG or steroids	Improved,	severe
Lisnic V. et. al./ Moldova	27/M	15 hours	HIV	Paresthesia and numbness in legs and right arm, spastic tetraparesis, urinary retention, constipation, T7 superficial and C7 deep sensory level	WBC: < /5mm^3 Protein: normal (number not reported) Glucose: normal; *** SARS-CoV-2 PCR: neg	Neg	Neg	NA	Spinal cord MRI revealed an extensive C4-T5 lesion mainly in posterior columns and right lateral column without gadolinium enhancement	Acute Transverse Myelitis	IVMP x 5 days followed by PLEX x 5 days	Improved	Non-severe
Zachariadis A. et. al./ Switzerland	63/M	12 Days	Obesity	Moderate paresis in lower limbs,	WBC: 16/mm^3 Protein: 57.3mg/dl	NA	NA	NA	Brain and Spine MRI reported as normal	Acute Transverse Myelitis	IVIG x 5 days followed by 5-	Partial Improvement	Non-severe

pyramidal signs, sensory level at T10.	Glucose: 34; *** SARS-CoV-2 PCR: neg	day steroid therapy

** =CSF glucose not available. *** = serum glucose not available. Abs, Antibodies; EVD, External Venous drainage; CSF, Cerebrospinal fluid; IVIG, Intravenous immunoglobulin; IVMP, Intravenous methylprednisone; ADEM, Acute disseminated encephalomyelitis; PRES, Posterior Reversible encephalopathy syndrome; LETM, Longitudinal extensive transverse myelitis; PLEX, Plasmapheresis; AHNE, Acute hemorrhagic necrotizing encephalitis; CLOCC, Cytotoxic lesion of the corpus callosum; MERS, Mild encephalitis/ encephalopathy with reversible splenial lesion; AHLE, Acute hemorrhagic leukoencephalitis.

Table S2. Studies with data from case reports and case series on COVID-19 associated PNS manifestation.

Author/ country	Patient age /gender	Time duration from COVID -19 to neurological symptom onset	Co-morbidity	Neurological presentation	CSF findings	Serum Anti gangliosides antibodies	MRI findings	Diagnosis/ Variant	Management	Outcome	Severity
Ottaviani D. et.al /Italy	66/F	7 Days	HTN	Paraplegia, areflexia	CSF: WBC$<5/mm^3$, Total Protein: 108mg/dL Glucose: *** glucose: **	Negative	NA	AIDP	IVIG x 5 days	Did not improve	Severe
Pfefferkorn T. et. al./ Germany	51/M	12 days	NA	Tetraparesis, Areflexia	CSF: WBC: 9cells/mm^3 Total protein: Normal Glucose: **; ***	Serum Anti-ganglioside antibodies negative	MRI spine massive symmetrical contrast enhancement of the spinal nerve roots at all levels of the	AIDP	IVIG x 5 days, PLEX x 14 sessions	Partial improvement	Severe

							spine including the cauda equina, anterior and posterior nerve roots				
Scheidl E. et. al. /Germany	54/F	21 days	None	Paresis of lower extremities, areflexia and paresthesia	CSF: WBC <5/mm3 Protein 140g/L Glucose: **. ***	NA	MRI cervical spine without contrast normal	AIDP	IVIG x 5 days	Improved	Non-severe
Hutchins K.L. et. al. /USA	21/M	16 Days	None	Facial weakness, dysarthria, diffuse areflexia	CSF: WBC <5/mm3 Glucose, 65mg/dl; *** Protein, 49mg/dl	NA/serologies were negative except for serum HSV, IgG and IgM	Abnormal enhancement of bilateral CN VII, CNV VI, Right CN III	BFP	PLEX x 5 cycles Denied IVIG for religious reasons	Improved	Non-severe
Arnaud S. et.al. / France	64/M	22 Days	DM type 2	Paraparesis, areflexia, paresthesia's	CSF: WBC: <5/mm3 Glucose: **; *** Protein 160mg/dl PCR: negative for SARs-CoV-2	Viral serologies are negative	NA	AIDP	IVIG x 5 days	Improved	Non-severe
Su X.W et.al. /USA	72/M	6 Days	CAD, HTN, Alcohol use disorder	Paresthesia's Quadriplegia, areflexia, sensory loss distal to knees B/L	WBC: 1 cell/mm^3 Protein: 313mg/dl Glucose: **; *** SARS-CoV-2 PCR: neg	Anti-ganglioside GM1, GD1b, and GQ1b and acetylcholine receptor binding, voltage-gated calcium channel, antinuclear, and anti-neutrophil cytoplasmic antibody titers were negative.	NA	AIDP	IVIG x 6 days	Did not improve	Severe
Riva, N. et.al/ Italy	60/M	17 Days	None	Progressive limb weakness, Paresthesias	WBC: <5/mm3 Protein: normal Glucose: **; ***	Anti-ganglioside antibodies: Negative,	MRI cervical spine was unremarkable	AIDP	IVIG x 5days	Partial improvement	Non-severe

				, facial diplegia, hypophonia dysarthria	SARS-CoV-2 PCR: neg	SARS IgG positive					
Otmani H. et. al. / Morocco	70/F	3 Days	RA	Paresthesia, Quadriplegia, ,	WBC<5/mm3 Protein 100mg/dl SARS-CoV-2 PCR: neg	NA	NA	AMSAN	IVIG x 5days	No significant improvement.	Non-severe
Camdessanche. J.P., et. al. /France	64/M	7 Days	None	Paresthesia, Tetra paralysis	WBC: <5/mm^3 Protein: 166mg/dl Glucose: **; ***	Anti-gangliosides antibodies: negative	N A	AIDP	IVIG x 5 days	NA	Severe
Caamaño D.S.J et al. /Spain	61/M	10 Days	NA	Bilateral facial nerve palsy	WBC: <5/mm^3 Protein 20mg/dl Glucose: **; ***	NA	MRI brain was unremarkable	BFP	Low dose prednisone	Partial improvement	Non-severe
Webb S. et. al. /UK	57/M	7 Days	HTN	Paresthesia Difficulty walking	WBC: <5/mm^3 Protein: 51mg/dl Glucose**; *** SARS-CoV-2 PCR: neg	anti-ganglioside antibodies were negative.	NA	AIDP	IVIG x 5 days	Did not improve	Severe
Assini A et. al. / Italy	55/M	20 Days	None	Bilateral ptosis, dysphagia, dysphonia, bilateral paralysis of hypoglossal nerve, 10th CN palsy	WBC: <5/mm^3 Protein was normal Glucose **; ***. Oligoclonal bands in CSF, increased IgG ratio 233 SARS-CoV-2 PCR: neg	Antiganglioside antibodies negative	NA	MFS	IVIG x 5 days	Improved	Non- severe
Assini A et. al./Italy Pre-print	60/M	20 Days	None	Right Foot drop, dysautonomia	WBC: <5/mm^3 Protein was normal. Glucose: **; ***	Antiganglioside antibodies: negative	NA	AMSAN	IVIG x 5 days	Improved	Severe

					Oligoclonal bands in CSF, increased ratio IgG/albumin: 170						
Toscano, G. et. al. /Italy	77/F	7 Days	None	Flaccid areflexic tetraplegia evolving to facial weakness, upper limb paresthesia, and respiratory failure	WBC 4/mm^3, protein level, 101 mg/dl Glucose: **; *** SARS-CoV-2 PCR: neg	Negative Anti-ganglioside antibodies	Spine: enhancement of caudal nerve roots	ASMAN	IVIG x 2, 2nd cycle of IVIG was ineffective 7 days after first cycle	Partial improvement	Severe
Toscano, G., et. al. /Italy	23/M	10 Days	None	Facial diplegia and generalized areflexia evolving to lower limb paresthesia with ataxia	WBC<5mm^3 protein level, 123 mg/dl; Glucose: **, *** SARS-CoV-2 PCR: neg	NA	MRI enhancement of facial nerve bilaterally Spine: normal	ASMAN	IVIG	Partial improvement	Non-severe
Toscano, G. et. al. /Italy	55/M	10 Days	None	Flaccid tetraparesis and facial weakness evolving to areflexia and respiratory failure	WBC<5/mm^3 Protein level, 193 mg/dl; Glucose: **; *** SARS-CoV-2 PCR: neg	Negative Anti-ganglioside antibodies	Head: normal Spine: enhancement of caudal nerve roots	AMAN	IVIG x 2 cycles	Poor outcomes	severe

Toscano, G. et. al. /Italy	76/M	5 Days	None	Flaccid areflexic tetraparesis and ataxia	WBC: <5/mm^3 Normal protein level; Glucose **; *** SARS-CoV-2 PCR: neg	NA	Head: normal<br>Spine: normal	AIDP	IVIG	Partial Improvement	Non- severe
Toscano, G., et. al. /Italy	61/M	7 Days	None	Facial weakness, flaccid areflexic paraplegia and respiratory failure	WBC: 3/mm^3 protein level, 40 mg/dl; Glucose: **; *** SARS-CoV-2 PCR: neg	positive SARS-CoV-2 IgG Negative Anti-ganglioside antibodies	Head: not performed<br>Spine: normal	AIDP	IVIG, PLEX	Partial Improvement	Severe
Dinkin, M. et. al. / USA	36/M	4 Days	Infantile strabismus	L ptosis, diplopia, B/L leg paresthesia, L oculomotor palsy, B/L 6 CN palsy, and hypoesthesia, Gait ataxia	NA	A ganglioside antibody panel was negative	Enhancement T2-hyperintenistym enlargement of L oculomotor nerve.	MFS	IVIG x 3 days	Improved	Non- severe
Dinkin, M. et. al. / USA	71/F	2 Days	NA	Diplopia, unable to abduct Right eye	WBC: 5/mm^3 Protein: normal Glucose: **; ***	NA	Enhancement of the optic nerve sheaths and posterior Tenon capsule	6th Nerve palsy	No IVIG or PLEX	Improved	Non-severe
Gutierrez-Ortiz. et. al. /Spain	50/M	5 Days	Bronchial Asthma	Areflexia, Broad based gait, right internuclear opthalmoparesis, Right fascicular oculomotor palsy	White count: 0 Protein 80mg/dl Glucose 62mg/dl SARS-CoV-2 PCR: neg	. Antibodies to gangliosides (GM1, GM2, GM3, GD1a, GD1b, GD3, GT1a, GT1b, GQ1b, and anti-sulfatide antibodies) in the serum were examined. The patient was	NA	MFS	IVIG x 5 days	Improved	Non- severe

						only positive for the antiGD1b–immunoglobulin G (IgG) antibody. T					
Gutierrez-Ortiz. et. al. /Spain	39/M	3 Days	None	Areflexia, severe abduction deficit in both eyes, fixation nystagmus, impaired upper gaze	White count: 2/mm^3(all monocytes) Protein 62mg/dl Glucose 50mg/dl, ***	negative serologies, including the rRT-PCR for SARSCoV-2	NA	Polyneuritis cranialis	None	Improved	Non-severe
Sedaghat K. et. al. / Iran	65/M	10 Days	DM type 2	Quadriplegia / Bilateral facial palsies /areflexia, Reduced vibration and fine touch sensation	CSf analysis could not be performed due to lack of consent	NA	Normal finding except for mild herniation of two intervertebral discs. (level is not reported)	AMSAN	IVIG x 5 days	Partial improvement	Non-severe
Zhao H et. al. /China	61/F	1 day		Areflexia, motor weakness, reduced sensation to touch and pin prick distally	CSF: WBC<5/mm^3, glucose: **; *** Protein: 124mg/dl	NA	NA	AIDP	IVIG (duration of therapy not reported)	Improved	Non-severe
Virani, A. et. al. /USA	54/M	8 days		Areflexia, decreased lower extremities followed by ascending paralysis, difficulty breathing	NA	NA	Thoracic and Spine MRI was normal	AIDP	IVIG x 5 days	Partially improvement	Severe
Alberti P et. al. /Italy	71/M	3 days	HTN, Abdominal aortic aneurysm, lung cancer s/p resection	Tetraparesis, Areflexia, hypoesthesia at distal limbs, dyspnea, low back pain	CSF: WBC: 9 /mm^3 unspecified cell type. Protein 54mg/dl Glucose: **; ***	NA	NA	AIDP	IVIG x 5 days	Deceased	Severe

Padroni, M. et. al. / Italy	70/M	24 Days	NA	Asthenia, hand and feet. Gait difficulty, Areflexia	WBC <5 /mm^3 Protein: 48mg/dl Glucose: **; ***	NA	NA	AIDP	IVIG x 5 days	Poor outcome	Severe
Coen M. et. al. / Switzerland	70/M	6 days	None	Bilateral lower limb paralysis, Areflexia	CSF: WBC<5/mm3 Protein levels not reported Glucose: **; ***	antiganglioside antibodies were not detected	No reported lesions on MRI brain	AIDP	IVIG x 5 days	Partially improvement	Non-severe
Mozhdehipanah H. et. al. /Iran	38/M	21 days	NHTN	bilateral facial paralysis and mildly dysarthric speech, areflexia, decreased sensation to all modalities in distal four limbs	WBC: <5mm^3 Protein: 139mg/dl Glucose: **; ***	NA	NA	AIDP	PLEX x 5 sessions	Improved	Non-severe
Mozhdehipanah H. et. al. /Iran	55/F	26 days	COPD	Acute progressive lower limb weakness, Areflexia, decreased pinprick, vibration in distal limbs	WBC: <5mm^3 Protein: 57mg/dl Glucose: reported normal; ***	Brain MRI reported as Normal	NA	AMSAN	IVIG x 5 days	Deceased	severe
Mozhdehipanah H. et. al. /Iran	66/F	30 days	DM type 2, HTN, RA.	Areflexia, decreased weakness in lower extremities, decreased sensation to light touch, position, vibration	WBC<5mm^3 Protein: 89mg/dl Glucose: reported normal, ***	NA	NA	AIDP	IVIG x 5 days	Partial improvement	Non- Severe

Tiet M.Y. et. al. /UK	49/M	21 days	None	Limb paresthesia, ascending limb weakness, facial diplegia,	WBC: <5mm³ Protein: 125mg/dl SARS-CoV-2 PCR: neg	NA	NA	AIDP	IVIG x 5 days	Improved	Severe
Embrahimzadeh, AS et. al. /Iran	46/M	18 days	None	peripheral facial nerve palsy on the right side, flaccid paralysis	WBC<5mm³ Protein: 78mg/dl, glucose 70mg/dl, ***	Gq1b antibody: non-reactive	NA	AIDP	No treatment	Improved	Non-severe
Embrahimzadeh, AS. et. al. /Iran	65/M	10 days	None	Areflexia, lower extremity weakness	NA	Gq1b antibody: non-reactive	NA	AIDP	IVIG x 1 course	Improved	Non-severe
Chan M et. al. /USA	68/M	18 days	None	bilateral facial weakness, dysphagia, dysarthria, neck flexion weakness, and inability to ambulate	WBC:<5mm³ Protein: 226mg/dl Glucose: 56mg/dl; *** SARS-CoV-2 PCR: neg	ganglioside antibody testing was unremarkable	MRI of lumbosacral spine was unremarkable	AIDP	PLEX x 5 sessions	Improved	Non-severe
Chan M. et. al. /USA	84/M	23 days	None	Proximal upper and lower limb weakness and diminished vibration and proprioception at the toes	WBC< 5/mm³ Protein: 67mg/dl Glucose: 58mg/dl: *** SARS-CoV-2 PCR: neg	elevated GM2 IgG/IgM antibodies	NA	AIDP	PLEX x 5 sessions, followed by IVIG	Partially improvement	Severe
Bigaut K. et. al. /France	43/M	21 days	None	Decreased sensation, flaccid paralysis in lower limbs	WBC<5/mm³ Protein: 94mg/dl Glucose: **; *** PCR for Covid-19: neg	Antiganglioside antibodies were negative	multiple cranial neuritis (in nerves III, V, VI, VII, and VIII), radiculitis, and plexitis on both the brachial and lumbar plexus	AIDP	IVIG x 5 days	Improved	Non-severe

Bigaut, K. et. al./France	70/F	10 days	None	Flaccid tetra paresis, generalized areflexia, forelimb paresthesia; respiratory failure	WBC<5/mm^3 Protein: 160mg/dl Glucose: **; ***	Negative	NA	AIDP	IVIG x 5 days	Improved	Non-severe
Chan JL et. al. /Canada	58/M	20 days	NA	facial diplegia and areflexia in the lower extremities	WBC<5/mm^3 Protein: 100mg/dl Glucose: **; *** SARS-CoV-2 PCR: neg	NA	Bilateral intracranial and extracranial facial nerve enhancement	AIDP	IVIG x 5 days	Partial Improvement	Non-severe
Helbok, R et. al. / Austria	68/M	10 days	None	Decreased sensation to touch and pinprick in the lower extremities, absent ankle jerk, and inability to walk	WBC <5/mm^3 Protein: 64mg/dl Glucose: **; ***	Antiganglioside antibodies	MRI Lumbar unremarkable findings	AIDP	Started with IVIG but switched to PLEX next day x 4 sessions	Partial improvement	Severe
Kilinc D et. al. /Netherlands	50/M	26 days	None	Facial diplegia, , mild symmetric proximal muscle weakness.	WBC < 5mm^3 Protein: Normal Glucose: **; *** SARS-CoV-2 PCR: neg	Anti-GQ1b was negative.	MRI brain Normal	AIDP	IVIG x 5 days	Partial improvement	Non-severe
Lantos J.E. et. al. /USA	36/M	2 days	None	Opthalmoparesis (including initial left CN III and eventual bilateral CN VI palsies), ataxia, and hyporeflexia.	NA	Asialo GM1 ganglioside antibody: equivocal Anti-GQ1b was negative	MRI Orbit: striking enlargement, prominent enhancement, and T2 hyperintense signal of the left cranial nerve (CN) III	MFS	IVIG (duration not reported)	Partial improvement	Non-severe

				Paresthesia in bilateral legs.							
Lascano A.M. et. al. /Switzerland	52/F	15 days	None	Back pain, limb weakness, ataxia, distal paresthesia, dysgeusia,. respiratory failure, dysautonomia and tetraplegia with areflexia	WBC 3 cell/μl; Protein level 60 mg/dl; Glucose: **, ***	Anti-ganglioside antibodies were negative	MRI spinal cord: Unremarkable	AIDP	IVIG x 5 days	Improved	Non-sever
Lascano A.M. et. al. /Switzerland	63/F	7 days	DM type 2	Lower limb pain, mild weakness and normal deep tendon reflexes. Developed tetraparesis, distal paresthesia and areflexia	WBC 2 cell/μl; Protein level 40 mg/dl; Glucose: **, ***)	NA	NA	AIDP	IVIG x 5 days	Partial improvement	Non-severe
Lascano A.M. et. al. /Switzerland	61/F	22 days	None	Lower limb weakness and distal paresthesia, dizziness, dysphagia, dysautonomia, areflexia. Presented worsening of bulbar symptoms and	WBC 4 cell/μl; Protein level 140 mg/dl; Glucose: **, *** SARS-CoV-2 PCR: neg	NA	Spinal cord: Lumbosacral nerve root enhancement Brain: Normal	AIDP	IVIG x 5 days	Partial improvement	Non-severe

				bilateral facial palsy							
Reyes-Bueno A. et. al. / Spain	51/F	15 days	None	Paresis of the left external rectus muscle with horizontal diplopia when looking to the left, discrete predominantly inferior bilateral facial paresis, symmetrical paraparesis in leg muscles and global areflexia.	WBC<5/mm^3 Protein: 70mg/dl Glucose: **; ***	Antiganglioside antibodies: negative	Unremarkable	MFS	IVIG x 5 days	Partial improvement	Non-severe
Sancho- Saldaña A. et. al. /Spain	56/F	15 days	None	Bilateral facial nerve palsy, dysphagia and severe proximal tetraparesis with global areflexia	WBC<5/mm^3 Protein: 86mg/dl Glucose: **; ***	Antiganglioside antibodies: Negative	MRI of whole spine: brainstem and cervical meningeal enhancement (up to C7 level)	AIDP	IVIG x 5 days	Partial improvement	Severe
Agosti E. et. al. / Italy	68/M	10 Days	Dyslipidemia, AAA, BPH, HTN	Facial diplegia, Paraplegia and bilateral ankle areflexia	WBC<5/mm^3 Protein: 98mg/dl Glucose: **; ***	NA	NA	AIDP	IVIG x 5 days	Improved	Non-severe
Lampe A. et. al. / Germany	65/M	3 days	None	Paresis of right arm and paresis of lower limbs, hyporeflexia,	WBC: <5mm^3 Protein: 56mg/dl Glucose: **; ***	ganglioside antibodies (GM1, GM2, GM3, GD1a, GD1b, GT1b, GQ1b) were negative	NA	AIDP	IVIG x 5 days	Improved	Non-severe

Manganotti P et. al. / Italy	50/F	16 days	None	Ataxia, ophthalmoplegia with diplopia in vertical and lateral gaze, left upper arm cerebellar dysmetria, generalized areflexia, mild lower facial defects	WBC <5mm³ Protein 74.9mg/dl Glucose: **; ***	anti-GM1, anti-GM2, anti-GM3, anti-GD1a, anti-GD1b, anti-GT1b, and anti-GQ1b negative	No abnormalities on MRI brain	MFS	IVIG x 5days	Improved	Non-severe
Guilmot A. et. al. /Belgium	37/NA	10 days	NA	Unilateral facial nerve palsy, hemifacial paresthesia, bilateral hearing loss and paresthesia in lower limbs.	WBC: 101/mm³ (95% lymphocytes) Protein 51mg/dl Glucose: **; ***	Anti-GD1b IgG titer: > 1/100	Thickened and abnormally hyperintense III cranial nerves enhancement, abnormal bilateral enhancement of cranial nerve V and abnormal bilateral enhancement of VI and VII. Lumbar spinal cord showed abnormal periconal enhancement of the pia-mater together with clumping and enhancement of the roots of the horse tail.	Facial palsy	IVMP, 64mg x 7 days	Improved	Non-sever
Guilmot A. et. al. /Belgium	40/NA	5 days	NA	Partial left oculomotor nerve III palsy	WBC<5mm³ Protein: 36mg/dl Glucose: **; ***	NA	MRI brain was normal	3rd nerve palsy	No treatment	Improved	Non-severe

Christiana Franke et. al. / Germany	67/M	NA	NA	Oculomotor disturbance	WBC<5mm^3 Protein: 336mg/dl Glucose: 113mg/dl; ***	Oligoclonal bands were positive in csf and serum	NA	3rd nerve palsy	NA	NA	NA
Diez-Porras L et. al. / Spain	54/M	5 Days	HTN, Obesity	Asymmetric paresis in both upper limbs, hypoesthesia both hands, global areflexia, bilateral facial palsy and dysphagia	WBC: <5mm^3 Protein: 52mg/dl Glucose: **; ***	Antiganglioside antibodies were measured in serum, obtaining IgM for GM2 and GD3 and a weak IgG band for GT1b.	NA	AIDP	IVIG however developed reaction and was stopped	Improved	Severe
Senel M. et. al. /Germany	61/M	15 days	NA	Ataxia, ophthalmoplegia, and general areflexia, Paresthesia on fingertips	WBC<5mm^3 Protein: 1588mg/dl glucose quotient was within normal range (0.51)	Anti-ganglioside antibody, Anti GQ1b: Negative	NA	MFS	IVIG x 5 days	Improved	Non-severe
Khaja M. et. al. /USA	44/M	3 days	HTN, Asthma	Facial diplegia	WBC: <5mm^3 Protein: 92 mg/dl Glucose: 77mg/dl; ***	IgG antibodies to GQ1B was negative	MRI brain and C spine: normal	Bilateral facial nerve palsy and AIDP	IVIG x 5 days	Improved	Non-severe
Abbaslou M.A. et. al. / Iran	55/F	26 days	Unknown chronic lung disease	Paresis in bilateral lower limbs, paresthesia in lower limbs, Areflexia	WBC: <5mm^3 Protein: 48.4mg/dl Glucose: 78mg/dl; ***	NA	NA	AMSAN	IVIG	Deceased	Severe
Defabio A.C et. al. / USA	70/F	90 days	Reflex sympathetic dystrophy, fibromyalgia,	Paresis in lower limbs, paresthesia in distal lower limbs,	WBC: 8/mm^3 Protein: 127mg/dl Glucose 79mg/dl; ***	NA	NA	AIDP	IVIG x 5 days	Improved	Non-severe

			GERD, hiatal hernia, asthma	Areflexia in both lower limbs.						
Nanda S et. al. / India	55/F	10 days	DM, HTN, Cholelithiasis	Paresis in all four limbs, areflexia	WBC: 5 cells, all lymphocytes Protein: 54mg/dl Glucose: 114mg/d; ***	NA	MRI of spine Degenerative changes in spine No Cord changes No nerve root enhancement	AMAN	IVIG x 5 days	Improved	Non-severe
Nanda S et. al. / India	72/M	6 days	HTN	Generalized hypotonia, paresis in both lower limbs, Areflexia	WBC: 0 Protein: 74mg/dl Glucose:110mg/dl; ***	NA	MRI of spine Mild degenerative changes, no cord changes, no nerve root enhancement	AIDP	IVIG x 5 days	Deceased	Severe
Nanda S et. al. / India	55/M	5 days	DM, HTN, CKD on hemodialysis	Generalized hypotonia, Paresis in both lower limbs, and areflexia s, glove and stocking sensory loss,	WBC: 5 cells, all lymphocytes Protein: 84mg/dl Glucose:94mg/dl; ***	NA	MRI of spine mild degenerative changes, no cord changes, no contrast enhancement	AMSAN	IVIG x 5 days	Improved	Non- severe
Nanda S. et. al. / India	49/M	10days	HTN	Bilateral facial palsy, Paresis in both lower limbs, Areflexia in both lower limbs	WBC<5mm^3 Protein: 52mg/dl Glucose:54mg/dl; ***	NA	MRI of spine mild degenerative changes	AIDP	IVIG x 5 days	Improved	Non-severe
Gigli G.L. et. al. / Italy	53/M	55 days	None	Lower limb paresthesia and paraparesis with ataxia, areflexia	WBC<5mm^3 Protein: 193mg/dl Glucose: **; *** SAR-CoV-2 CSF IgG, IgM pos	GM1, GM2, GM3, GM4, GD1a, GD1b, GD2, GD3, GT1a, GT1b, GQ1b) negative	Mri brain Unremarkable	AIDP	IVIG x 1 cycle	Improved	Non-severe
Zito, A. et. al. / Italy	57/M	17 days	NA	Paresis of lower limbs, loss of touch and	WBC<5mm^3 Protein: normal, no oligoclonal bands	Anti-GM1, anti-GD1b, and anti-GQ1b IgG	NA	AMSAN	IVIG x 5 days	Improved	Non-severe

				vibration in feet and ankles, gait ataxia, ankle areflexia and hyporeflexia in other limbs	Glucose: **; ***	and IgM were negative					
Korem S. et. al. /USA	58/F	14 days	Anterior Cervical discectomy and anterior interbody arthrodesis at C3-C7	Paresthesia, Bilateral lower extremity paresis, ataxia, hyporeflexia	WBC<5mm^3 Protein: 117mg/dl Glucose: **, ***	NA	MRI lumbar spine without contrast showed moderate bilateral and moderate left-sided neural foraminal narrowing at L2–L3 and L3–L4, respectively, and unremarkable conus medullaris. MRI Brain: Normal	GBS/ Subtype unspecified, NCS not performed	IVIG x 4 days	Improved	Non-severe
Garnero M. et. al./ Italy	65/M	NA	NA	NA	WBC<5mm^3 Protein: NA Glucose:	Anti-gangliosides antibodies: Negative	NA	AIDP	IVIG	Improved	Non-severe
Garnero, M. et. al./ Italy	73/M	0 days	NA	NA	WBC<5mm^3 Protein: 60mg/dl Glucose:	Anti-gangliosides antibodies; Negative	NA	AIDP	IVIG	Improved	Non- severe
Garnero, M. et. al./ Italy	55/M	20 days	NA	NA	WBC<5mm^3 Protein: 30mg/dl Glucose:	Anti-gangliosides antibodies: negative	NA	MFS	IVIG	Improved	Non-Severe
Garnero, M. et. al./ Italy	46/F	3 days	NA	NA	WBC<5mm^3 Protein: 100 g/dl Glucose:	Anti-gangliosides antibodies: Negative	NA	AIDP	IVIG	Partial improvement	Severe
Garnero, M. et. al./ Italy	60/M	20 days	NA	NA	WBC<5mm^3 Protein: 20mg/dl Glucose:	Anti-gangliosides antibodies: negative	NA	AMSAN	IVIG	Partial Improvement	Severe

Garnero, M. et. al./ Italy	63/F	15 days	NA	NA	WBC<5mm3 Protein: 90mg/dl Glucose:	Anti-gangliosides antibodies: Negative	NA	AMSAN	IVIG	Partial Improvement	Severe
Masuccio F.G et. al. /Italy	70/F	15 days	Obesity, HTN	Tetraparesis, paresthesia in both lower limbs with urinary retention and perineal areflexia, hyperreflexia in all limbs.	WBC<5mm3 Protein: Normal, Oligoclonal band mirror pattern Glucose: **; ***	Anti-GD1b IgM: positive	MRI brain normal. MRI spine showed hyperintensity in the posterior portion of the spinal cord and encompassed two vertebral levels (C7-T1), and non-enhancing.	AMAN	PLEX followed by IVIG.	Partial improvement	Non-severe
Galán, A.V et. al. / Spain	43/M	10 days	NA	Symmetric paresis and paresthesia in all four limbs, global areflexia, bilateral facial palsy, dysphagia	NA	NA	NA	AIDP	IVIG x 5 days	Partial improvement	Non-severe
Pelea T et. al. /Germany	56/F	19 days	HTN, Hypothyroidism	Tetraparesis, Areflexia, reduced vibration in bilateral stocking pattern	WBC: 9/mm3 Protein: 575mg/dl Glucose:67.3;104mg/dl	Anti-ganglioside antibodies (GM1-, GQ1b-antibodies) were absent	MRI of spine was normal	GBS/AMAN	PLEX x 7 sessions followed by IVIG x 5 days	Partial improvement	Severe
Kopscik M.R. et. al. /USA	31/M	Unclear	None	CN VI-VII and XII palsies, dysmetria, Gait ataxia, patellar and achilles areflexia.	WBC<5mm3 Protein: Normal Glucose: **; ***	Presence of anti-ganglioside – GQ1b (Anti-GQ1b) immunoglobulin G antibodies (1:100)	MRI brain and lumbar spine Normal	GBS/MFS	PLEX followed by IVIG (duration of therapy is not reported)	Partial improvement	Non- severe
Wada S. et. al. /Japan	69/M	17 Days	DM	Hyporeflexia, paralytic ileus, Areflexia,	WBC<5/mm3 Protein: 202mg/dl Glucose: **, ***	NA	NA	GBS (variant not specified)	IVIG x 5 days	Improved	Severe

				paresis of limbs						
Abrams R.M.C. et. al./USA	67/F	10 days	Breast cancer	Progressive quadriparesis, Paresthesias, urinary retention, global areflexia, Left facial and bulbar weakness	WBC<5mm^3 Protein: 222mg/dl Glucose:61mg/dl; ***	Ganglioside, acetylcholine receptor, lyme, and HIV antibodies were negative	MRI brain and spine unremarkable	GBS (variant not specified)	PLEX x 2 sessions	Partial improvement	Severe
Hirayama T. et. al./Japan	54/F	20 days	Asthma	Symmetric distal paresis, Paresthesia in all limbs, areflexia in lower limbs	WBC<5mm^3 Protein: Reported Normal Glucose: **; ***	Antiganglioside antibodies- GM1, GM2, GD1a, GD1b, GD3, GalNAc-GD1a, GT1a, GT1b, GQ1b and GA1 (asialo-GM1) were negative	NA	GBS (variant not specified)	None	Improved	Non-severe
Oguz-Akarsu E. et. al./ Turkey	53/F	3 days	NA	Mild dysarthria, bilateral lower limb paresis and paresthesia, Areflexia in lower limbs	WBC<5mm^3 Protein: 32.6 mg/dl Glucose: **; ***	NA	MRI of the lumbar and cervical spines revealed asymmetrical thickening and hyperintensity of postganglionic roots supplying the brachial and lumbar plexuses in STIR sequences.	GBS/AIDP	None	Improved	Non-severe
Homma Y. et. al. /Japan	35/F	2 days	NA	Facial paralysis, anosmia	WBC<5mm^3 Protein: 17mg/dl Glucose:61mg/dl;***	NA	NA	Facial nerve palsy	None	Improved	Non-severe
Gogia B. et. al. / USA	58/M	5 days	COPD, HTN, CAD, Facial trauma	Decreased sensation in V1-V3 distribution (CN V), Left sided facial palsy	NA	NA	MRI brain Normal	NA	Valacyclovir x 7 days	Improved	Non-severe

Looy E.V. et. al. / Belgium	53/F		GBS x 2 times, CIPD, (IVIG every 6 weeks)	Paresthesia in all limbs, areflexia in ankle, gait ataxia	WBC<5mm^3 Protein: 100mg/dl Glucose: 79mg/dl; ***	NA	NA	GBS/ Exacerbation of CIDP	IVIG x 5 days	Partial improvement	Severe
Bastug A. et. al./ Turkey	66/M	5 days	NA	Paresis in lower limbs, areflexia in lower limbs, bilateral hypoesthesia in lower limbs	WBC: 0 Protein: 233.5mg/dl; Glucose: **; ***	NA	MRI brain normal	GBS (variant not specified)	IVIG, followed by PLEX	Deceased	Severe
Mostel Z. et. al. /USA	69/F	1 month	HTN, HLD, Sickle cell trait, iron deficiency anemia, stroke (30 years ago with residual left sided weakness)	Proximal weakness in left upper and lower limbs, Left sided paresthesia in arms and legs, areflexia in ankle	NA	NA	MRI of the brain showed chronic microvascular ischemic changes. MRI of the spine revealed abnormal enhancement in the cauda equina	GBS/AIDP	IVIG x 5 days	Improved	Severe
Tekin A.B. et. al./Turkey	34/F	16 Days	Nulliparous, 37th gestational week	Paresis in lower limbs, right sided facial paresis, paresthesia in extremities, global areflexia	WBC: 0 Protein: 62.34mg/dl Glucose: normal; ***	NA	NA	GBS/AMSAN	IVIG x 5 days	Improved	Non-severe
Boru U.T. et. al. / Turkey	35/M	18 days	None	Dysautonomia, weakness in lower limbs, Hyporeflexia	WBC<5mm^3 Protein: 50mg/dl Glucose: **; ***	NA	NA	GBS/ acute motor axonal neuropathy	None	Improved	Non-severe
Zubair, A.S. et. al. /USA	32/M	65 days	None	Lower limb paresis and	WBC<5mm^3 Protein: 127.6mg/dl Glucose: **; ***	Negative for GM1 and GD1a/b antibodie	NA	GBS/ AMSAN	IVIG (duration not reported)	Partial improvement	Non-severe

				paresthesia, ankle areflexia						
Zubair, A.S. et. al. /USA	61/M	60 days	DM, severe lumbar stenosis, right foot drop,	Mild proximal upper limb paresis, moderate lower limb paresis, ataxia, areflexia	WBC<5mm^3 Protein: 54mg/dl Glucose: **; ***	No ganglioside antibodies.	NA	GBS/AMSAN	IVIG (duration not reported)	Improved	Non-severe
Aasfara J. et. al. /Morocco	36/F	42 days	Pregnant 37 weeks gestation	Hyporeflexia in lower limbs, Grade 3 nystagmus with bilateral facial palsy	WBC<5mm^3 Protein: 80mg/dl Glucose: **; ***	Anti-ganglioside antibodies were negative	Brain and spine MRI reported as normal	GBS/AIDP Bifacial weakness and paresthesia Right vestibulocochlear neuritis	IVIG x 5 days	Partial improvement	Non-severe
Mcdonnell E.P. et. al. / USA	54/M	3 days	Type 2 DM,	Facial diplegia, paresthesia and dysphagia, dysarthria, paraparesis, urinary retention	WBC<5mm^3 Protein: 74mg/dl Glucose:69mg/dl; Serum 109mg/dl	Anti GM1 ganglioside, IgM and IgG: negative	NA	GBS (variant not specified)	IVIG x 5 days	Improved	Non-severe
Abolmaali M. et. al./Iran	88/F	2 days	HTN	Neck flexion weakness, quadriparesis, areflexia in lower limbs	WBC<5mm^3 Protein: 88mg/dl Glucose: **, ***	NA	NA	GBS/AMSAN	PLEX x 6 sessions	Partial improvement	Severe
Abolmaali M. et. al. /Iran	47/M	10 days	NA	Dysarthria, generalized hyporeflexia, urinary retention, quadriparesis	WBC 0 Protein: 154mg/dl Glucose: **, ***	NA	MRI brain and whole spine reported as normal	GBS/AMSAN	PLEX x 2 session	deceased	Severe

Abolmaali M. et. al. /Iran	58/M	9 days	NA	Muscle weakness, ataxia, areflexia	WBC 0<br>Protein: 65 mg/dl<br>Glucose: **, ***	NA	NA	GBS/AMSAN	IVIG x 2 days then switched to PLEX due to high Creatine	deceased	Severe
Bueso T. et. al./ USA	60/F	22 days	Migraine	Bilateral lower limbs paresis and paresthesia	WBC 0<br>Protein: 197mg/dl<br>Glucose: **, ***	NA	NA	GBS (variant not specified)	IVIG x 5 days	Improved	Non-severe
